# The Objective Structured Clinical Examination (OSCE) in Periodontology with Simulated Patient: The Most Realistic Approach to Clinical Practice in Dentistry

**DOI:** 10.3390/ijerph20032661

**Published:** 2023-02-01

**Authors:** Gema Cidoncha, Marta Muñoz-Corcuera, Virginia Sánchez, María Jesús Pardo Monedero, Ana Antoranz

**Affiliations:** 1 Department of Clinical Dentistry, Faculty of Biomedical and Health Sciences, Universidad Europea de Madrid, 28670 Madrid, Spain; 2 Department of Preclinical Dentistry, Faculty of Biomedical and Health Sciences, Universidad Europea de Madrid, 28670 Madrid, Spain

**Keywords:** dental education, OSCE, evaluation, standardized patient, periodontics

## Abstract

The objective structured clinical examination (OSCE) is becoming an increasingly established assessment test in dental schools. The use of simulated patients in the OSCE makes the stations more similar to clinical practice. Therefore, the student can show their technical and clinical knowledge, and certainly, their ability to manage the patient. These sorts of tests, in which simulated patients can be included, would be used before the student started clinical practice with patients and/or at the end of the degree. The objective of this work was to describe how the periodontology station was developed using a simulated patient for students of a fifth year dentistry degree taking an OSCE test. Furthermore, a questionnaire was created to learn the perception of the students about this station and its characteristics. The fifth year students at the European University of Madrid positively evaluated this station in their examination. In addition, it was recorded that they preferred a simulated patient in their tests, rather than stations with clinical cases, images, X-rays, and presentations. It is essential that once the OSCE has been completed, the student receives a feedback to learn where they have failed and, therefore, be able to improve any of the aspects evaluated in the station.

## 1. Introduction

The OSCE or objective structured clinical examination is a practical test that assesses clinical competencies in a planned and structured way. Compared with traditional examinations, it is more objective [1,2]. It was introduced in the late 1970s by Harden et al. [3] with the intention of assessing competencies in relation to students’ skills, knowledge, and attitudes in different situations. Over the last few years, this kind of test has been fully integrated into the study programmes of medicine, dentistry, and others health care degrees. Nowadays, it is the gold standard for competency skills tests [4].

### 1.1. OSCE Structure

There is a long and complex process for designing and implementing an OSCE. To carry out its implementation, it is necessary to understand the educational principles of this type of test and to develop academic and administrative structures. Once it has been implemented, it is important to perform periodically quality controls and constant improvements to maintain the test’s psychometric precision [5].

The OSCEs in health degrees consist of stations [6]. These are defined as simulated clinical scenarios with specific objectives, in which the different competences and skills of the students are evaluated, such as the diagnosis and management of patients in a standardized way [4]. Students go through the different stations, performing the specific clinical task in a simulated environment, which involves interaction with objects or patients, whether they are real or simulated ones [7]. The number of them varies according to what is being evaluated and the difficulty of the test; the stations are between 8 and 10 min long. All of the students perform the OSCE under the same conditions to standardize the test and enable the results to be compared in an objective manner [4]. Each station is hold by one or two assessors who observe and evaluate the student’s performance in the specific task. There are standardized correction tables with predetermined criteria and checklists, and they have between 10 and 30 items with different calibrated scores, which are used to evaluate the test [4,7].

To compare the OSCE to traditional exams, it is not intended to measure the acquisition of knowledge per se, but it will determine the student’s ability to synthesize information and apply clinical knowledge, allowing the examiners to judge their diagnostic expertise and clinical decision making [8].

### 1.2. Simulated Patient

Often some of the stations are carried out with simulated patients, with whom the students must interact [9]. In these situations, the student’s knowledge can be assessed, as well as their communication skills with the patient [5]. Both actors and non-teaching staff linked to the university can perform as simulated patients [7]. In either situation, simulated patients are trained to accurately play the role that has been developed and use scripts that have been established for each of the stations. The lines must be complete and detailed to maintain the objectivity of the test [4]. In addition, it will always be necessary to guarantee the security and confidentiality of the scripts [7].

Simulated patients should behave in the same way with all of the students, including the emotions they should express during the test. That will provide important information, not only for the evaluation of the patient’s examination skills, but also interpersonal skills. Therefore, it is necessary to prepare and train the actors to standardize their participation [10].

### 1.3. OSCE in Dentistry

In 2017, the European University of Madrid (UEM) began to use this mode of evaluation. The OSCE has been used worldwide in medical education for more than 40 years. Recently, it has begun to be introduced in dental schools, where it remains a less frequently used test compared to the frequency of its use in other health sciences degrees [10,11].

Conventionally, the most used form of assessment to determine the knowledge and understanding of dental students is the multiple choice written test. This type of exam is easy to design, review, and grade. However, in a written test cannot evaluate the students’ clinical competences [12].

Given the shortcomings of standard examinations and the special characteristics of dental training, it is necessary to explore other assessment tools, above all, in the transition from preclinical to the clinical practice. For this reason, the OSCE was implemented in dentistry [12,13].

In this field, the OSCE can be created according to the clinical subject areas, such as: conservative areas, periodontology, prosthesis, etc. In order to develop a model of skill groups and repeated measures, the academic degree’s objectives can be used. The aims of the OSCE in dentistry are based on evaluating the competencies of students in relation to knowledge skills, procedures, clinical reasoning, anamnesis, communication (verbal and written), the promotion of oral health (education), and their interaction with patients [14].

A few publications exist on the use of simulated patient in periodontology using the OSCE approach. The justification for this project lies in the fact that, traditionally, the methods used in periodontology assessments have been either multiple choice exams or case studies. Usually, a student is shown a case study with a relevant oral disease or pathology and required to provide the examiners with a diagnosis and course of treatment, either verbally or in writing. In the case of this paper, an OSCE-style evaluation has been chosen, which not only represents a new method of assessment—including simulated patients that interact with the student—but it also provides feedback on the evaluation method from the students through the use of surveys [15]. The use of the simulated station seeks to go beyond simply diagnosing and recommending a course of treatment, but instead, also to observe the student’s interactions with the patient. A checklist and/or overall rating scale is used by the reviewers in order to evaluate the students’ competences, and ideally, they would provide written feedback. In the present study, this role has been undertaken by the simulated patient. They are professional actors who are trained to perform this job, which allows the OSCE results to contribute greatly to progress and improve the different exams models, and thus, it would make the reliability of the examiners’ judgement more important [16].

The purpose of this work was to describe the set-up of the periodontology station with a simulated patient, which took place in the fifth year of a dentistry degree. In addition, a questionnaire was created to learn the perception of the students about this station and its characteristics.

## 2. Materials and Methods

Approval was granted by the UEM Ethics Board, and it was assigned the internal code CIPI 22.238.

The periodontology station was used by fifth grade dentistry students. At the European University of Madrid, the students combine theory lectures with clinical practice. There are some pathologies which are rare to see in the university dental practice, therefore, the periodontology teachers decided to design an OSCE station which showed a real-life situation, even though it was not very common at the university. Furthermore, they contemplated that the pathology should be interesting, easy to diagnose—if diagnostic criteria are known—and simple to evaluate because of its low complexity of treatment. 

They selected necrotizing gingivitis (NG) as the pathology that had to be evaluated in the periodontology OSCE. As the disease is associated with pain and bad breath, it was decided to carry out the station with the collaboration of a professional actor, who could interact with the student, expressing the symptoms verbally. A 21-year-old actress with specific personality was selected.

The simulated patient had to look like a person with good personal care. So, she was asked to arrive groomed, neat, and well dressed. She was asked to be apparently very calm and to come with books, a school bag or some materials that made it seem that she was studying. However, despite this appearance, she had to be very concerned about knowing in detail what was happening to her, being insistent, asking questions and trying to understand the pathology she suffered. Her main characteristic was hypochondria, something she had to show to the students.

The patient should explain that she was taking medication for anxiety. This was due to the stress of her university studies, exams, and the little time she had. She marked a pain level of 8 on a visual analog scale (VAS) of 1–10. She was asked to express that she did not want to be touched during that visit as she could not stand it. She was instructed to say that she only wanted an assessment and an explanation, making it clear that it would not be treated during that visit, and thus, the teachers would be able to assess the students’ reaction and management of this situation.

Regarding the case history of the simulated patient, considerations were established about her medical and dental history, other than just describing the disease using the signs (through X-rays and photographs) and symptoms described by the patient.

The patient’s medical history included a family background of periodontitis and a personal record including varicella and appendicitis surgery. She was a patient who smoked 10 cigarettes per day, and she was without allergies or other pathologies of interest. She was prescribed 1 mg Lorazepam and instructed to take a quarter of a pill every day before going to bed.

In respect to her current disease, she was undergoing dental treatment. Fillings were made in a dental practice, and she attended an emergency appointment after experiencing deep gum pain for 5–6 days and bad breath.

In an intraoral examination, the patient had gum inflammation with the presence of pseudomembranous lesions and an alteration of the gingival anatomy in the interdental papillae. There was lots of plaque and bleeding. To support the case, a real necrotizing gingivitis patient’s radiography was provided, and a document with the anamnesis, background, patient’s dental complain, and signs and symptoms was also supplied to the students in case the need to check it at any time. 

The patient was given a script to follow, and prior training was carried out to clarify her doubts and establish a behaviour pattern that was faithful as possible to the reality and the established text, which consisted of the following points:

She had a mother with periodontitis, but she did not know if her condition was related to it or if she suffered from the same problem, and she asked about the differences. Once the student explained the diagnosis of necrotizing gingivitis, she was asked to insist on the question of whether it could be necrotizing periodontitis and to enquire for information about the pathology.

She was asked to describe having bad breath and requested to insist on knowing why. She should inquire if she ought to perform some special cleaning and if she could use a rinse that she had at her mother’s house, without remembering the composition of it.

At the end of the station, the actor had to rate the student based on a checklist in relation to: communication skills, diagnostic judgment, therapeutic judgment, prevention, and ethical and legal considerations. For each of these items was assigned a score according to their degree of importance in the station, with the highest being 3 and the lowest a 1, representing a total of 47 points, with the student needing at least 50% of the total score to pass (Table 1).

This test was carried out in April 2022 by fifth year UEM students of dentistry. It was consisted by 5 stations that lasted for 8 min each.

In July 2022, an online questionnaire was carried out. A total of 29 students who had completed the periodontology station took part. All of them participated voluntarily and signed an informed consent form. This survey aimed to learn the perception of students about the use of a simulated patient in the periodontology OSCE.

The survey was designed using Microsoft Office Forms. The questions were differentiated into general sociological data (age, sex, and prior studies), and five specific questions with multiple choice answers about the periodontology station were used, in which the student had to select a single answer of the four that were proposed. Finally, an open-ended question was included, so that each student could contribute their specific considerations about this station (Table 2).

Statistical analysis: The answers were exported in a Microsoft Excel sheet, which was the software used for the analysis. A descriptive statistic of the participants’ sample was made. The absolute and relative frequencies referring to each answer option in each of the questions were calculated.

## 3. Results

A total of 29 students in their fifth year of a degree in dentistry took part in the study. All of them signed the informed consent form. There were 37.93% males and 62.06% females. The mean age of participants was 25.68 ± 1.96 years.

The answers obtained from the survey are shown below (Table 3, Table 4, Table 5 and Table 6).

Most of the students (58.62%) thought that the patient’s management was difficult, even though they were able to perform the test. No students expressed being unable to handle the patient.

Additionally, 65.51% of the students thought that this situation could happen in real dental practice. Oddly, there were three participants who considered that the situation it could not happen in real life. 

Most of the surveyed students felt that the use of simulated patient for the OSCE evaluation was more similar to the reality than other options would be such as cases studies, photographs, or radiographies. 

In addition, the management of the patient’s anxiety was chosen by the participants as the most complicate part of the station, followed by stablishing a correct diagnosis, treatment plan, and finally, planning the check-ups. 

Regarding the open question in which students were invited to express their opinion about the station with a simulated patient, the answers were positive in all of the cases. There are some examples such as: “It was perfect”, “Very good”, “The periodontology OSCE was the closest to the real practice”, “It was a very interesting experience, it reflected reality”, and “It approaches to a situation that could happen during a real practice, it is very good to train and know what we have to improve in our day to day”. 

## 4. Discussion

Unlike other exam formats that dental students are more familiar with, OSCE provides the opportunity to apply knowledge in real-world scenarios. From an educational point of view, the OSCE allows them to think critically, integrate their knowledge, and prepare for the next phases of their education, which are appreciated by the students [1]. In fact, the OSCE could be especially used for the dental students’ training and to evaluate their skills before starting internships in a clinical environment [1]. There are studies in which the OSCE has been used as a predictive method to learn about the ability of the students in a clinical environment, since these situations cannot be assessed through traditional written tests [11,17]. In addition, by observing the students during the OSCE, the instructor will obtain information on how to guide them in the skills that are taught during their university education, and according to the results obtained from the test, the teachers can analyse and assess any weak areas and develop plans to improve upon them [11,17].

Since the OSCE has long been established in dentistry, it is important to learn the student’s perception about it in relation to the educational value of the method and if they are able to promote important skills, such as the application of knowledge or problem solving [1]. Both in the study of Graham et al. [1], as in our case, the students valued the OSCE test highly, since they were able to show their clinical skills. The main disadvantage that the students reported in relation with the OSCE, as compared with a preclinical practical exam, is the anxiety generated by the uncertainty of each station [1,18]. Wu et al. [19] evaluated the degree of anxiety of 226 dental students who were taking their first OSCE type test, in which they were evaluated communication skills and the use of medical history and different clinical procedures. The results indicated that most of the students showed a moderate degree of anxiety before the test, with it being slightly higher in women. In the present study, the students’ anxiety was not measured, but in the open question, they recorded that the patient’s anxiety made them especially nervous. 

When one is evaluating the knowledge about periodontology through an OSCE, the tendency in the scientific literature has been to use clinical images or a periodontal chart that has already been made, and the student interprets what they could observe from them. Other authors such as Manogue et al. [20] developed the periodontology OSCE, including scaling and root planning and the teaching of oral hygiene techniques. However, by the UEM’s periodontology teaching staff, it was decided to go a step further and introduce an OSCE station with a simulated patient with particular characteristics (nervousness and anxiety). The two targets of introducing the simulated patient were to be able to evaluate the behavioural management of the student towards the patient, as well as the competences of the diagnosis, treatment, and management of the NG. 

In order to implement the new periodontology station, it was necessary to find an actress who would act as a patient and create a script that she could master. The actress was instructed to control the scenario, simulate signs and symptoms of NG, answer medical history questions, and provide information about complementary tests, if it was requested. As NG can occur with a lot of pain, she was asked to act with a nervous and anxious attitude, always in a similar way with each of the students. The teaching staff decided that once the OSCE had been completed, it should be the actress who had to evaluate the student. The actress was provided with and given training for the evaluative checklist of this periodontology station, which consisted of 15 questions with a yes or no answer options, which had been modified from prior studies [20,21,22]. The items that had to be completed by the actress dealt with the NG itself, as well as the management of her nervous attitude on the part of the student. The checklist was integrated into the UEVALUA digital platform, which is the tool through which UEM students are graded during their clinical practices with patients. The platform provides, as terms of its main virtues, the standardization of the evaluations, and once they are qualified, the student has access to the grade and can see each of the items that have been evaluated. Therefore, through UEVALUA, as in Manogue’s study [20], grade feedback is established between the teachers and the students; the student can see where they have failed and how they can improve for the future. The concept of feedback was valued very highly by the students, since the comments and items valued by the teachers could help them a lot in their academic training [20].

One of the main features of the present study is the involvement of a simulated patient, who at the same time, was the reviewer. Regarding the simulated patient in dentistry and its difficult implementation, it has been compared with other studies accomplished at a dental school where the OSCE was performed through a virtual reality system. In this respect, Donn et al. [23] tested a virtual OSCE assessment (VOSCE) for a dental undergraduate, and they provided a comprehensive overview of how to design and apply it.

By referring to the examiners, the literature shows us that the teachers, and not the simulated patients, conducted the follow-up on the students [24]. In some cases, a highly protocolized calibrations is carried out. Yeates et al. [25] developed a station where an examiner’s pre-calibration was made. For this purpose, a video of a student’s exam was showed, so each assessor could evaluate it independently, which allowed them to standardize the evaluation criteria for each of the participating teachers. 

Once the periodontology station of the OSCE was carried out in the UEM, a questionnaire was undertaken by the students about the perception of it, emphasizing the use of a simulated patient. The students at the UEM, as well as those consulted in the publications of Schoonheim et al. [18] and Manogue et al. [20], felt more positive about using the station with the simulated patient than those in which there were clinical cases, presentations with images or X-rays. However, the students consulted by Shahzad [26] and Park [12] did not show any statistically significant difference in their satisfaction with the stations with simulated patients compared to those with simulated clinical or practical cases. Although the satisfaction of the teachers with this test was not measured at the European university, it is significant that when one is scoring the general enthusiasm of the teachers and students, higher averages were obtained for teachers, scoring their satisfaction on a scale from 1 to 5, thus obtaining better rating compared with those of the students. 

The main difficulty facing UEM student at the periodontology OSCE was the management of the patient and the diagnosis of the NG. The survey was answered by fifth grade students, who had two years of clinical practice with patients. In the questionnaire, they recorded that having experienced clinical practice with numerous patients had helped them to manage the patient’s state of nervousness and anxiety. In addition, 65.51% of the students considered that this kind of patient and situation could happen in real dental practice. These results are particularly significant for UEM teaching staff, as they insist from the beginning of clinical practices on the importance of the behavioural management of the patient to gain their confidence and to be able to carry out the established dental treatment.

Although simulated patient OSCE stations have numerous advantages compared to those of other stations and even other assessment methods, there are several logistical factors that make their implementation difficult. They are stations that require a lot of preparation: the selection and instruction of the actor, planning, and rating system calibration, arranging a large space with several rooms, employing support staff to use the computer, and developing the complementary tests that may be needed (study models, X-rays, periodontal chart, etc.). 

This study has some limitations. There were only 29 students who were included, which makes it difficult to compare these results to a wider population. Therefore, the sample size would be larger in the future, covering the opinion of both the teachers and actors as well. 

The actor received training on the student’s evaluation based on a checklist, which had previously been developed by the teachers. The aim of this was to provide an objective and reliable evaluation. However, the assessment might turn out not to be accurate. In subsequent studies, it should be determined if this kind of test works under a teacher’s observation who evaluates the student independently, and thus, verifies the evaluations’ coherence.

## 5. Conclusions

The OSCE is an evaluation method that is increasingly being used in dental schools. In addition, using simulated patients in the stations gives the option of being able to assess the trainee’s technical, clinical, and behavioral knowledge in front of the patient.

The students who participated in the satisfaction questionnaire evaluated the experience of the periodontology station with a simulated patient as positive. Most of them were able to attend to the patient in a situation that could happen in real dental practice. On the other hand, the most complicated thing for them consisted of managing the patient’s anxiety, which was rated above establishing the diagnosis or treatment plan of the pathology she presented.

The students surveyed reflected that they found it more similar to clinical practice when the OSCE station used simulated patients than when other types of stations were used.

To build a patient station, many more resources are required, which must be considered, in comparison to those of more basic stations.

## Figures and Tables

**Table 1:** Station evaluated items checklist.

ITEMS	Questioned/Explored/Performed	Detailed Description	Competence	YES	NO	Score
1	Empathy, assertiveness	Greets me politely (shakes my hand, address me using “you”)	Communication skills			3
2	Assertiveness	Listens properly, does not interrupt the patient, looks at me while talking	Communication skills			2
3	Respect	Does not make criticisms or pejorative comments to the patient	Communication skills			3
4	Respect and interest	Is interested in the opinions, concerns, or emotions of the patient’s parent	Communication skills			2
5	Diagnosis	The student informs me that the pathology is necrotizing gingivitis (NG)	Diagnostic Judgment			3
6	Medical history, stress and anxiety	Tells me that stress influences the development of this pathology	Diagnostic Judgment			3
7	Medical history, stress and anxiety	Discuss that stress causes immunosuppression and therefore favors the appearance of this pathology	Diagnostic Judgment			1
8	Medical history and epidemiology	Discloses that sex does not influence the development of pathology	Diagnostic Judgment			1
9	Medical history, stress and anxiety	The student comments that anxiolytic medication does not affect the development of the disease	Diagnostic Judgment			3
10	Symptoms and diagnostic signs	Explains clearly what the pain and bad breath are due to	Diagnostic Judgment			3
11	Symptoms and diagnostic signs	Informs me clearly why the papillae are not right	Diagnostic Judgment			2
12	Treatment	Explains to me that scale (prophylaxis) is the treatment of choice	Therapeutic Judgment			3
13	Treatment	Postpone the prophylaxis for a week due to severe pain	Therapeutic Judgment			3
14	Treatment	Prescribes me rinses with Chlorhexidine 0.12% every 12 h until the next appointment	Prevention			3
15	Antibiotic therapy	I am prescribed Metronidazole 250 mg × 2 every 8 h	Therapeutic Judgment			3
16	Differential diagnosis	Knows and explains the difference between gingivitis and necrotizing gingivitis	Diagnostic Judgment			3
17	Differential diagnosis	Explains what it is necrotizing periodontitis and why this is not	Diagnostic Judgment			3
18	Informed consent	Give the patient the IC of periodontics treatment	Ethical and legal			3

**Table 2:** Survey and multiple choice answers.

There was a simulated patient in the periodontology station, do you think that having completed two years of internship with patients at the university dental practice could help you manage the patient’s anxiety?
o I attended to the patient without problem. o Although she was a difficult patient, I was able to assist her. o It was difficult for me to manage this sort of patient. o I was not able to handle the patient.
2. The periodontology station was carried on with a simulated patient. Do you think this kind of pretended scenario could happen in real practice?
o The situation arranged at the station was not real. o There are no patients with such a difficult attitude in real dental practice. o There is no such complex periodontal pathology in real dental practice. o The situation shown at the station could certanly take place in real dental practice.
3. What about the methodology that was used in the OSCE do you think is most similar to real dental practice and would make you better reflect your professional skills?
o Clinical case. o Simulated patient. o Clinical images of lesions. o Radiographies interpretation.
4. What do you consider to be the most complicated part of the periodontology station with the simulated patient?
o Anxiety management. o Treatment plan. o Correct diagnosis. o Planning periodontal check-ups.
5. Below, you can express your views regarding the periodontology station in a respectful manner.

**Table 3:** Answers to question 1. There was a simulated patient in the periodontology station. Do you think that having completed two years of internship with patients at the university dental practice could help you manage the patient’s anxiety?

	n	%
I attended to the patient without problem	10	34.48
Although she was a difficult patient, I was able to assist her	17	58.62
It was difficult for me to manage this sort of patient	2	6.89
I was not able to handle the patient	0	0

**Table 4:** Answers to question 2. The periodontology station was carried on with a simulated patient. Do you think this kind of pretended scenario could happen in real practice?

	n	%
The situation shown at the station could certainly take place in real dental practice	19	65.51
There are not patients with such a difficult attitude in real dental practice	5	17.24
The situation arranged at the station was not real	3	10.34
There is no such complex periodontal pathology in real dental practice	2	6.89

**Table 5:** Answers to question 3. What of the possible methodology to be used in the OSCE do you think would be more similar to real dental practice and would make you better reflect your professional skills?

	n	%
Clinical case	3	10.34
Simulated patient	21	72.41
Clinical images of lesions	4	13.79
Radiographies interpretation	1	3.44

**Table 6:** Answers to question 4. What do you consider to be most complicated of the periodontology station with the simulated patient?

	n	%
Anxiety management	11	37.93
Treatment plan	8	27.58
Correct diagnosis	9	31.03
Planning periodontal check-ups	1	3.44

## Data Availability

Not applicable.
